# Development of a yeast-based CRISPR genome editing system for feline coronavirus

**DOI:** 10.3389/fmicb.2025.1627509

**Published:** 2025-08-14

**Authors:** Xiaohu Zhang, Jingru Zhu, Di Zhang, Yueping Zhang

**Affiliations:** ^1^State Key Laboratory of Veterinary Public Health and Safety, College of Veterinary Medicine, China Agricultural University, Beijing, China; ^2^China Agricultural University Veterinary Teaching Hospital, Beijing, China

**Keywords:** feline coronavirus, feline infectious peritonitis, CRISPR, TAR, reverse genetics, virus rescue, yeast artificial chromosome

## Abstract

**Introduction:**

Feline infectious peritonitis (FIP), caused by feline coronavirus (FCoV), is a highly lethal disease characterized by systemic organ infection in cats. Current challenges of FIP include the absence of definitive diagnostic criteria, effective vaccines, and targeted therapies. Developing a robust genome editing toolkit is therefore critical to unraveling FCoV replication and pathogenesis mechanisms, elucidating viral protein functions, and identifying promising diagnostic and therapeutic targets.

**Methods:**

In this study, we developed a yeast-based CRISPR genome editing system compatible with a TAR-generated FCoV infectious clone, enabling gene deletion, gene insertion, and point mutation with high efficiencies and accuracies.

**Results and discussion:**

This system not only will contribute to a better understanding of the pathogenic mechanisms of FCoV but also serves as a valuable platform for vaccine development. Furthermore, it offers a possible strategy for genome editing and reverse genetics for other coronaviruses.

## 1 Introduction

Feline coronavirus (FCoV) is a non-segmented, enveloped, positive-sense single-stranded RNA virus in the family *Coronaviridae* and order *Nidovirales* (Gao et al., 2023). The spherical viral particles (80–120 nm in diameter) feature a helical nucleocapsid and a crown-like envelope with spike proteins (15–20 nm in height) (Jaimes and Whittaker, 2018). The 29 kb FCoV genome contains 11 open reading frames (ORFs): ORF 1a, ORF 1b, ORF S, ORF 3a, ORF 3b, ORF 3c, ORF E, ORF M, ORF N, ORF 7a, and ORF 7b. These encode two polyproteins (pp1a and pp1b) for replication, four structural proteins (spike protein S, membrane protein M, envelope protein E, and nucleocapsid protein N), and five accessory proteins (3a, 3b, 3c, 7a, 7b) critical for viral pathogenesis (De Barros et al., 2019).

FCoV is categorized into two biotypes based on clinical manifestations: feline enteric coronavirus (FECV) and feline infectious peritonitis virus (FIPV). FECV exhibits low virulence, typically causing mild or asymptomatic gastrointestinal infections through replication in intestinal epithelial cells. In contrast, FIPV is highly virulent, replicating in monocytes and macrophages to trigger systemic immune-mediated disease, manifesting as fatal dry or wet feline infectious peritonitis (FIP) with multi-organ involvement. Additionally, FCoV is divided into two serotypes based on host antibody profiles: FCoV-I and FCoV-II. FCoV-I is the predominant serotype in clinics, however, the type-I viruses perform poorly in laboratory cell lines and are hard to isolate and study. Conversely, FCoV-II is less prevalent in nature but replicates efficiently in commercially available Crandell-Rees feline kidney (CRFK) and *Felis catus* whole-fetus 4 (Fcwf-4) cell lines, making it a preferred model for experimental studies over the past decades (Dye and Siddell, 2005; Licitra et al., 2013; de Haan et al., 2005; Haijema et al., 2003; Haijema et al., 2004; Rottier et al., 2005; Tekes et al., 2012; Amer et al., 2012). The global rise in cat has elevated the epidemiological risks of FIPV, posing unprecedented challenges to veterinary public health systems. A stark example emerged in 2023 when Cyprus experienced a devastating FIPV outbreak attributed to the novel strain FCoV-23 (FCoV-II). This variant FCoV demonstrated an alarmingly high rate of FIP progression and mortality, leading to thousands of feline fatalities and causing huge economic losses (Warr et al., 2023; Attipa et al., 2023).

Establishing a reverse genetics system is essential for the in-depth investigation of FCoV invasion, replication, and pathogenesis. Several reverse genetics systems have been established for coronaviruses, including targeted RNA recombination (Haijema et al., 2003; Kusters et al., 1990), Bacterial Artificial Chromosome (BAC) vectors system (Almazán et al., 2000; Jin et al., 2021; Almazán et al., 2013; Nguyen et al., 2021; Bálint et al., 2012; Wang et al., 2021), *in vitro* ligation system (Yount et al., 2000; Fang et al., 2007; Lee et al., 2019; Scobey et al., 2013; Fahnøe et al., 2022; Gu et al., 2024), vaccinia virus vectors system, and transformation-associated recombination (TAR) cloning (Thi Nhu Thao et al., 2020; Cao et al., 2023). While these approaches enable the rescue of recombinant viruses, they are often inefficient for precise and rapid gene-editing. For instance, although TAR cloning allows genome reassembly with site-specific mutations through multi-fragment recombination, it requires rigorous validation of genome integrity and sequence accuracy due to the complexity of the assembly process. Therefore, the development of more efficient and accurate genome-editing systems remains crucial for advancing coronavirus pathogenesis research.

CRISPR-Cas9, a recently developed genome-editing tool, uses synthetic single-guide RNAs (sgRNAs) to direct Cas9 nuclease activity, inducing double-strand breaks (DSBs) at target DNA sequences. These breaks are repaired by the cell’s natural homology-directed repair system, enabling precise modifications such as mutations, deletions, insertions, or replacements of the target sequence (Jinek et al., 2012). This system has been widely adopted for gene editing in cells of divers species, revolutionizing the creation of transgenic animal models and advancing medical research. Our lab previously developed a gRNA-tRNA array for CRISPR-Cas9 (GTR-CRISPR) in *Saccharomyces cerevisiae* (*S. cerevisiae*) for rapid simultaneous editing of multiple genes (Gong et al., 2021).

In this study, we combined the TAR cloning with GTR-CRISPR system, enabling efficient genome editing (including gene deletions, insertions, and point mutations), and successfully rescuing genetically modified viruses. This approach offers a simple, rapid, cost-effective, and high-efficiency strategy for manipulating the FCoV genome.

## 2 Materials and methods

### 2.1 Cell culture and viral propagation

The CRFK cell line, maintained in our laboratory, was cultured in Dulbecco’s modified Eagle’s medium (DMEM; Sigma Aldrich, D6429) supplemented with 10% fetal bovine serum (FBS; Clark, FB25015) and 1% penicillin–streptomycin (PS). Cells were incubated at 37°C in a humidified 5% CO_2_ atmosphere.

### 2.2 Bacterial and yeast strains

Chemically competent *E. coli* DH5α (Tiangen Biotech) was used for CRISPR plasmid construction. Electrocompetent *E. coli* DH10B (Weidi Biotech) was used for plasmid amplification. DH5α was transformed via heat shock, and DH10B via electroporation. Transformed bacteria were cultured in LB broth and selected on LB agar plates containing appropriate antibiotics. *S. cerevisiae* CEN. PK113-5D (MATa *ura3-52, MAL2-8c, SUC2*), maintained in our laboratory, was transformed using the lithium acetate-PEG method. Yeast transformants were selected on synthetic complete dropout (SC-URA) agar plates with antibiotics.

### 2.3 Virus strains

The full-length genomic sequence of the rFCoV 79–1,146 strain was previously constructed. Briefly, 11 fragments were PCR-amplified from the cDNA of the commercialized 79–1,146 strain and assembled into a yeast artificial chromosome (YAC) shuttle plasmid via TAR in yeast. *E. coli* DH10B was used for amplification of the shuttle plasmid. Transfection of the resulting plasmid into CRFK cells successfully rescued the recombinant virus.

A T-to-C mutation was introduced at nucleotide position 121 of the 3c gene in the rFCoV 79–1,146 genome. This mutation converted the 41st codon from a TAG (stop codon) to CAG (encoding glutamine, Gln), restoring full-length expression of the previously silenced 3c gene. The rescued virus was named rFCoV 79–1,146-3c.

A 721-nucleotide deletion at nucleotide position 5–725 of the 3c gene was engineered in the rFCoV 79–1,146 genome, abolishing 3c protein expression. The remaining 4 nucleotides at the N-terminus of the 3c gene preserved the TGA stop codon for the upstream 3b gene and provided the ATG start codon for the downstream E gene, thereby maintaining the original reading frame. The resulting virus was designated rFCoV 79–1,146-3 cd.

A deletion spanning the 3a gene, 3b gene, and the first 240 codons of the 3c gene was introduced into the rFCoV 79–1,146 genome. This modification abolished 3a and 3b protein expression while retaining a truncated N-terminal 3c protein, with no disruption to the original reading frame. The rescued virus was named rFCoV 79–1,146-3abc.

The rFCoV 79-1146-DF infectious cDNA clone was engineered from the rFCoV 79–1,146 genome through a 338-nucleotide deletion targeting the C-terminal 132 nucleotides of the 3a gene, the entire 3b gene, and the N-terminal 50 nucleotides of the 3c gene. This modification resulted in the expression of a truncated C-terminal 3a protein, complete loss of 3b protein, and disruption of the 3c start codon coupled with an in-frame premature stop codon, thereby abolishing 3C protein expression. The rescued virus was designated rFCoV 79-1146-DF.

The 3a, 3b, and 3c genes in the rFCoV 79–1,146 genome were replaced with an enhanced green fluorescent protein (eGFP) cassette, enabling eGFP expression during infection. This modification allows real-time monitoring of viral replication via fluorescence microscopy. The rescued virus was designated rFCoV 79-1146-eGFP.

### 2.4 gRNA target design and pCas plasmid construction

**Figure 1:**
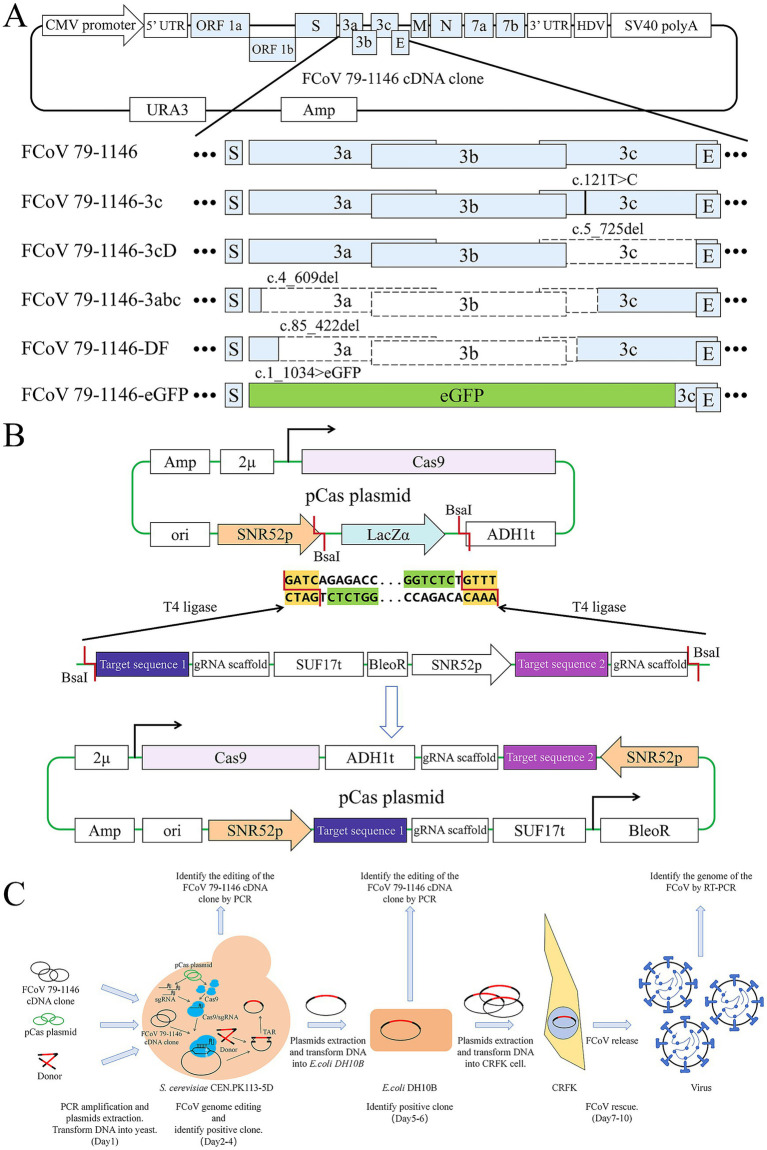
Strategy diagram of plasmid construction, virus editing and rescue process. **(A)** Elements of FCoV 79–1,146 infectious cDNA clone plasmid and it’s edited gene sequences. Black lines represent mutation sites, blank boxes represent deleted sequences, light green box represents the eGFP sequence. **(B)** Elements of pCas plasmid and the diagram of GoldenGate. The LacZ sequence on the original plasmid was cut down by *Bsa*I, and the DNA fragment containing the gRNA target sequence was seamlessly inserted to the plasmid by T_4_ ligase. **(C)** The full process of viral genome editing and virus rescue.

The pCas-lacZα plasmid, derived from prior studies (Figure 1B), carries a mutant Cas9 (D147Y and P411T) and a high-copy 2 *μ* origin of replication. The lacZα cassette, flanked by the *SNR52* promoter and gRNA scaffold, was excised using the *Bsa*I restriction enzyme. The lacZα region was replaced with a PCR fragment containing the gRNA target sequence, gRNA scaffold, *SNR52* promoter, and a selection marker. This PCR product was amplified from an intermediate plasmid containing the gRNA scaffold (with its terminator), a bleomycin resistance gene (BleoR, including promoter and terminator), and the *SNR52* promoter. Primers were designed with 5′ Golden Gate ligation sites, *Bsa*I recognition sequences, and 20-bp gRNA target sequences, ensuring a total primer length ≤60 bp.

gRNA target sequences were designed by identifying “NGG” protospacer adjacent motifs (PAMs) near editing sites using SnapGene, with the 20-bp region downstream of the PAM selected as the targeting sequence. All gRNA target sequences are listed in Table 1. Constructed pCas plasmids were named pCas-FCoV 79–1,146-3c, pCas-FCoV 79–1,146-3 cd, pCas-FCoV-3a3b3c, pCas-FCoV 79-1146-DF, and pCas-FCoV-eGFP.

**Table 1:** gRNA target sequences and donor primers.

Virus	Target Sequence(5′-3′)	dsDNA donor primers (5′-3′)
rFCoV 79–1,146-3c	3c-sgRNA:tgttatagtacaacagcatt	3c-dsDNAdonor-F: caacatgaaaatgttatagtacaacagcatcaggttgttagtgctagaacacaaaattat 3c-dsDNAdonor-R: ataattttgtgttctagcactaacaacctgatgctgttgtactataacattttcatgttg
rFCoV 79–1,146-3cD	3cD-sgRNA:tgttatagtacaacagcatt	3cD-dsDNAdonor-F: taaagagagattatagaaaaattgccattctaaattccatgcgaaaatgacgttccctag 3cD-dsDNAdonor-R: aacaaccatgccatggtcatctatgatagtaaatgccctagggaacgtcattttcgcatg
rFCoV 79–1,146-3abc	3abc-sgRNA-1:ttccatgcgaaaatgattgg 3abc-sgRNA-2:ctacttgtgtgtataggtttg	3abc-dsDNAdonor-F: aataaattccttaagaactaaacttattagtcattacaggtcttgtatgtttaagattg 3abc-dsDNAdonor-R: ctataagcataggccctacaagtgtcattgatacaatcttaaacatacaagacctgtaa
rFCoV 79-1146-DF	3abc-sgRNA-1:ttccatgcgaaaatgattgg 3abc-sgRNA-2:ctacttgtgtgtataggtttg	DF-dsDNAdonor-F: tgtacttgacgaacttgaccgtgcatactttgctgtaactcttaaaccatgttattgtt DF-dsDNAdonor-R: atgttgtgtagtatgcacatttgctgtgttattaacaataacatggtttaagagttaca
rFCoV 79-1146-eGFP	eGFP-sgRNA:atgaacatcccgttcttacc	eGFP-50 bp-F:ccaattgaaaaagtgcatgtccac eGFP-50 bp-R:atgccctagggaacgtcatag eGFP-500 bp-F:gggtgtgatgtgttgtttgtcaa eGFP-500 bp-R:gtggtctgccatattgtaacactg

For Golden Gate assembly, a 20 μL reaction mixture contained: 2 μL 10 × T4 ligase buffer (M0202, New England Biolabs); 1.6 μL *Bsa*I (R0535, New England Biolabs); 0.4 μL T4 ligase (M0202, New England Biolabs); 348 ng pCas plasmid (8.7 kb); 60 ng PCR fragment (1.5 kb). The reaction protocol was as follows: 37°C for 30 min; 25 cycles of 37°C for 10 min and 16°C for 5 min; 16°C for 30 min; 37°C for 30 min; 80°C for 6 min; Hold at 4°C.

### 2.5 Viral genome editing

The infectious cDNA clone plasmid of FCoV 79–1,146 was transformed into *S. cerevisiae* and selected on synthetic complete dropout uracil (SCD-Ura) agar plates. Positive transformants were subsequently transformed with the plasmids pCas-FCoV 79–1,146-3c, pCas-FCoV 79–1,146-3 cd, pCas-FCoV-3a3b3c, pCas-FCoV 79-1146-DF, and pCas-FCoV-eGFP respectively, followed by secondary selection on SCD-Ura plates supplemented with 300 ng/mL bleomycin. Validated positive clones were confirmed via colony PCR to ensure accurate target editing.

For plasmid recovery, a single verified colony was inoculated into 10 mL of SCD-Ura liquid medium and cultured overnight at 30°C with 200 rpm shaking. The culture was then scaled up to 50 mL and incubated under the same conditions for 24 h. Yeast plasmids were extracted using the TIANGEN Plasmid Mini Kit according to the manufacturer’s protocol.

### 2.6 Plasmid transformation into *E. coli*

Plasmids extracted from yeast were transformed into *E. coli* DH10B electrocompetent cells. Positive transformants were selected on Luria-Bertani (LB) agar plates containing 25 ng/mL chloramphenicol. Single colonies were screened via colony PCR to confirm target editing accuracy. Verified clones were cultured overnight in 10 mL of LB liquid medium with 25 ng/mL chloramphenicol at 30°C and 200 rpm, then scaled up to 250 mL and incubated under the same conditions for 24 h. Plasmids were extracted using the TIANGEN Endotoxin-Free Plasmid Maxi Kit, concentrated via ethanol precipitation, validated by restriction enzyme digestion, and used for cell transfection.

### 2.7 Virus rescue

Plasmids were transfected into CRFK cells via electroporation. Cytopathic effects (CPE) were monitored until 50% of cells exhibited CPE, after which the culture underwent two freeze–thaw cycles to release virions. Cellular debris was removed by centrifugation to obtain Passage 0 (P0) virus strain. P0 strain was passaged once to generate P1 strain. Viral RNA was extracted using the Novizan Nucleic Acid Extraction Kit, treated with DNaseI to remove residual DNA, and reverse-transcribed into cDNA. Full-length viral genomes were amplified via PCR with specific primers and sequenced. P1 strain aliquots were stored at −80°C.

### 2.8 Indirect immunofluorescence assay (IFA)

CRFK cells were seeded in 24-well plates. At 90% confluency, virus were infected at an MOI of 0.1. After 2 h incubation, the inoculum was replaced with 2% FBS-DMEM. At 48 h post-infection, cells were fixed with 4% (v/v) paraformaldehyde for 15 min, permeabilized with 0.3% (v/v) Triton X-100 for 15 min, and blocked with 5% BSA for 1 h. Cells were incubated overnight at 4°C with rabbit anti-FIPV N antibody (1:100 dilution), followed by FITC-conjugated goat anti-rabbit IgG (1:200 dilution) for 1 h at 37°C in the dark. Nuclei were stained with DAPI for 15 min. Fluorescence was visualized using a microscope.

### 2.9 Western blot

At 48 h post-viral inoculation, cells were lysed with RIPA buffer (Solarbio) on ice for 30 min. Supernatants were mixed with 5X SDS loading buffer, denatured at 95°C for 10 min, and separated on a 12% SDS-PAGE gel. Proteins were transferred to a PVDF membrane, blocked with 5% skim milk for 1.5 h at room temperature, and probed overnight at 4°C with rabbit anti-FIPV N antibody (1:100 dilution). After TBST washes, membranes were incubated with HRP-conjugated goat anti-rabbit IgG (1:10000 dilution) for 1.5 h at room temperature. Signals were detected using chemiluminescence.

### 2.10 Viral stability assay

CRFK cells at 90% confluency were infected, respectively, with rFCoV 79-1146-eGFP of P1, generation. The expression of eGFP was visualized via fluorescence microscopy 24 h post-infection (hpi).

### 2.11 Drug protection assay

CRFK cells seeded in 12-well plates were infected with virus at an MOI of 0.1. After 1 h incubation, the inoculum was replaced with 2% FBS-DMEM containing serially diluted GS-441524 (dissolved in DMSO), alongside negative control (virus-only) and blank control (mock-infected) groups. Following 24 h incubation at 37°C with 5% CO_2_, cells were washed with PBS, detached using 1 mL of 0.25% trypsin–EDTA, and resuspended in PBS for flow cytometry. Cells were analyzed on a BD FACSCanto flow cytometer using the FITC channel to quantify GFP-positive cells. Voltage settings for FSC and SSC were adjusted in the Cytometer FACSCanto parameters to center the main cell population. A gate was defined to exclude debris and aggregate cells. Data from 10,000 events per sample were recorded. GFP-positive cell percentages were calculated using FlowJo software.

## 3 Results

### 3.1 Establishment of the CRISPR editing system for exogenous plasmid DNA

The complete workflow for CRISPR-mediated editing of the FCoV infectious cDNA clone is illustrated in Figure 1C, with all gRNA target sequences and donor primers listed in Table 1. Constructed pCas plasmids were sequenced to confirm the correct insertion of 20-bp gRNA sequences upstream of the gRNA scaffold. The infectious cDNA clone plasmid of FCoV 79–1,146 was transformed into *S. cerevisiae*, and positive transformants were selected. Subsequently, pCas plasmids and donor DNA were co-transformed into these yeast cells to facilitate genome editing. Edited plasmids were extracted from yeast, transformed into *E. coli* DH10B for amplification, and finally transfected into CRFK cells to rescue recombinant viruses.

### 3.2 FCoV genome editing

#### 3.2.1 Point mutation

To validate the CRISPR editing system, we first introduced a single nucleotide substitution (A122C) in the 3c gene of the parental FCoV 79–1,146 plasmid. This mutation was successfully incorporated into the infectious cDNA clone, generating FCoV 79–1,146-3c (Figure 1A). Both single-stranded DNA (ssDNA) and double-stranded DNA (dsDNA) donor templates (Table 1) were tested, demonstrating that *S. cerevisiae* efficiently utilizes these two kinds of donor to resolve DSBs induced by CRISPR-Cas9 via homology-directed repair (HDR).

#### 3.2.2 Gene knockout

To assess the knockout efficiency of the CRISPR system, distinct pCas plasmids and donor DNA templates (Table 1) were designed to target specific regions of the parental FCoV 79–1,146 plasmid for deletions of varying lengths (Figure 1A). A 735-bp deletion spanning the entire 3c gene was engineered to generate FCoV 79–1,146-3 cd, while a 609-bp deletion targeting the N-terminal regions of 3a, 3b, and 3c produced FCoV-3a3b3c. Additionally, a 338-bp deletion in the ORF3 locus was introduced to create FCoV 79-1146-DF, truncating 3a and 3c while deleting 3b. According to the results, both ssDNA and dsDNA donors facilitated precise homologous recombination of these deletions, demonstrating the system’s robust capability to resolve large-fragment genomic modifications. This approach confirmed the versatility of CRISPR-mediated editing in achieving targeted gene knockouts across diverse genomic regions.

#### 3.2.3 Gene replacement

Finally, we tried to use longer dsDNA Donor to guide the replacement of genes. The ORF3 locus was replaced with a 717-bp eGFP cassette to generate FCoV 79-1146-eGFP (Figure 1A). A dsDNA donor (purified PCR product, Table 1) facilitated seamless integration of the eGFP sequence, enabling real-time visualization of viral replication via fluorescence.

### 3.3 Plasmids validation and virus rescue

**Figure 2:**
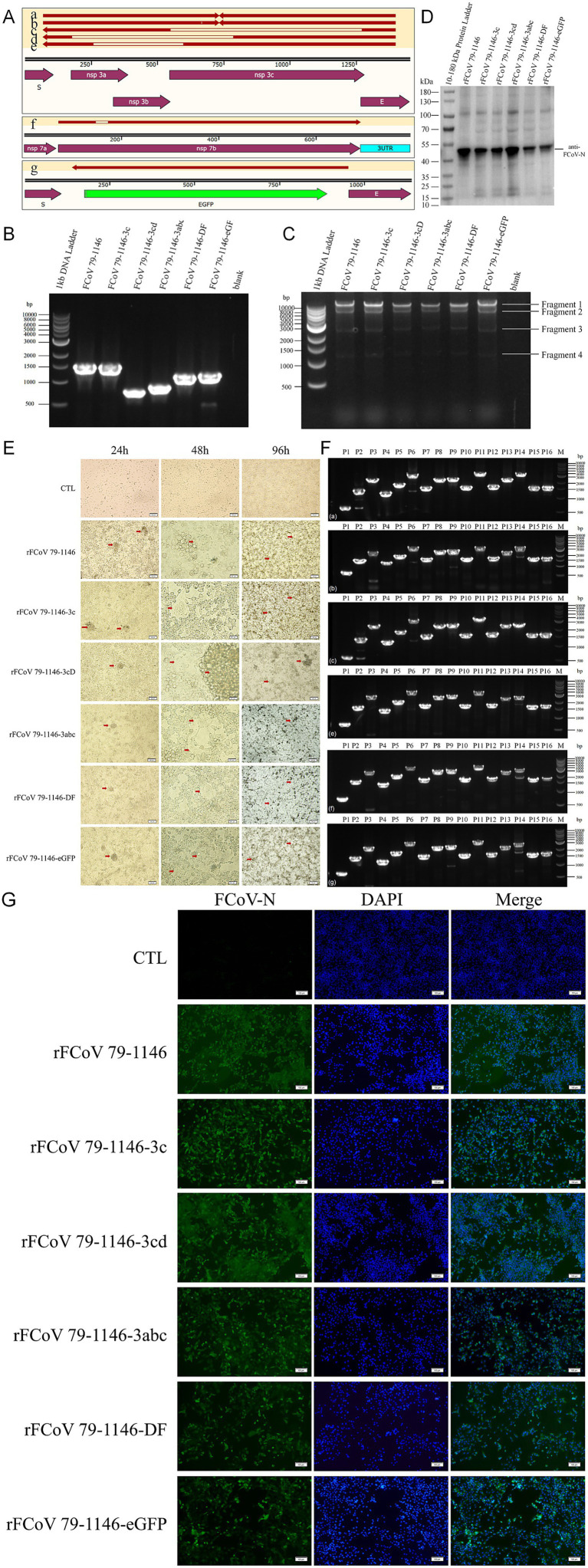
Edited plasmids verification and mutated virus recovery verification. **(A)** Sequencing and alignment of edited plasmids recovered from bacteria. **(B)** Identification of six plamids (FCoV 79–1,146, FCoV 79–1,146-3c, FCoV 79–1,146-3cD, FCoV 79–1,146-3abc, FCoV 79-1146-DF, and FCoV 79-1146-eGFP) edited regions by PCR. The theoretical molecular weights of PCR products are 1,450 bp, 1,450 bp, 729 bp, 841 bp, 1,112 bp, 1,136 bp, respectively. **(C)** Identification of SmaI digestive products of 6 plasmids (mentioned above) by PCR. The theoretical molecular weights of fragments 1 are 32,235 bp, 32,235 bp, 31,515 bp, 31,626 bp, 31,897 bp, and 31,921 bp, respectively. The theoretical molecular weights of other fragments are fixed, which are 7,876 bp, 2,842 bp, and 1,239 bp (fragment 2–4). **(D,F)** 6 recombinant virus (rescued by above plasmids) infected CRFK cells for 24 h was identified by IFA and WB. **(E)** Identification of fragment of 6 recombinant virus (rescued by above plasmids) genome by RT-PCR. The theoretical molecular weights of P14 are 3,159 bp, 3,159 bp, 2,439 bp, 2,550 bp, 2,821 bp, 2,845 bp, respectively. The theoretical molecular weights of other fragments are fixed, which are 641 bp, 1,461 bp, 2,439 bp, 1,259 bp, 1878 bp, 3,068 bp, 1,581 bp, 2,371 bp, 2,495 bp, 1,584 bp, 3,126 bp, 1,676 bp, 2,357 bp (P1-13), 1,592 bp, 1,639 bp (P15-16). **(G)** Significant CPE were observed at 48 and 96 hpi with CRFK cells.

Ten randomly selected positive yeast transformants were screened via colony PCR using primers flanking the donor homology arms. Electrophoresis revealed significant band shifts in PCR products compared to the parental plasmid, with sizes matching expectations. Sanger sequencing of PCR products confirmed 100% editing efficiency. Edited plasmids were transformed into *E. coli*, validated by colony PCR, and extracted using an endotoxin-free plasmid maxiprep kit (Tiangen Biotech). Target sequence PCR (Figure 2B), restriction enzyme digestion (Figure 2C), and PCR products sequencing (Figure 2A) confirmed the integrity and accuracy of edited plasmids, with no unintended insertions, mutations, or deletions, demonstrating the system’s high precision and stability.

**Figure 3:**
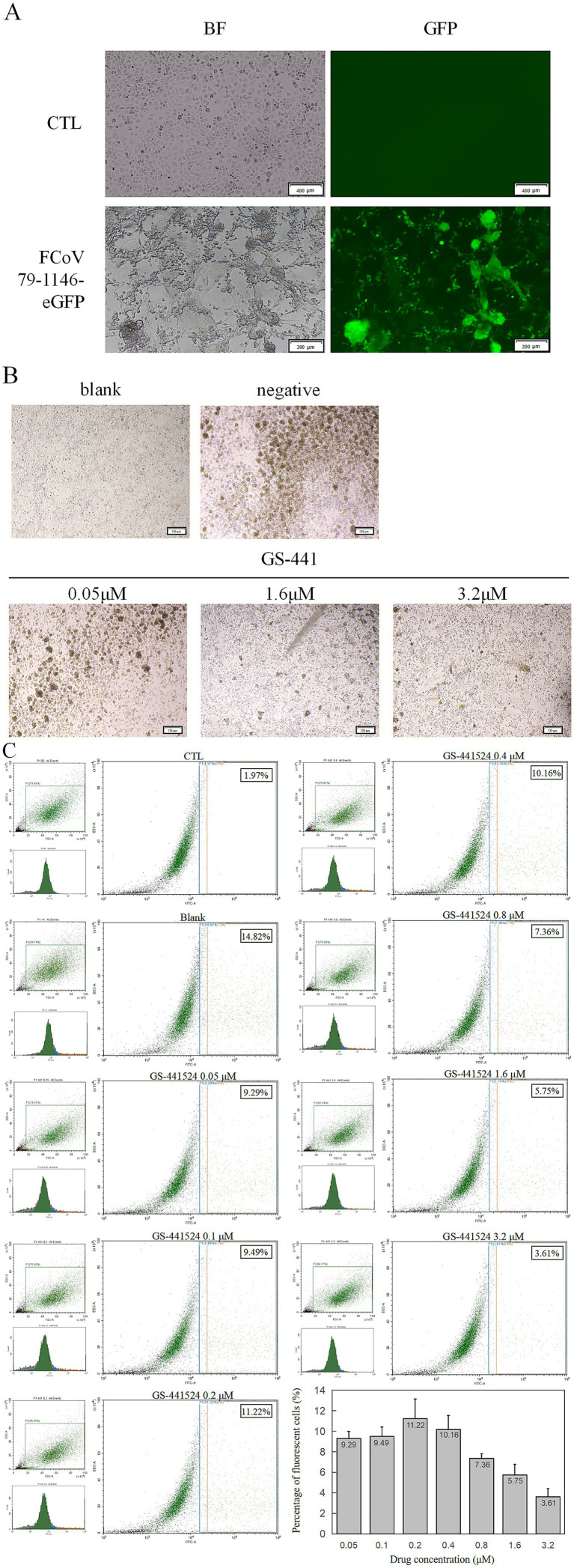
Fluorescence validation and antiviral protection of rFCoV 79-1146-eGFP. **(A)** Significant green fluorescence can be observed at 48 hpi with CRFK cells. **(B)** CPE decreased significantly with the concentration of GS-441524 increased. **(C)** Flow cytometer results after co-incubation of GS-441524 with rFCoV 79-1146-eGFP. At GS-441524 concentration of 0.05 μM, 0.1 μM, 0.2 μM, 0.4 μM, 0.8 μM, 1.6 μM and 3.2 μM, the proportion of fluorescent cells was 9.29, 9.49, 11.22, 10.16, 7.36, 5.75, 3.61%.

Following plasmid transfection into CRFK, CPE including cell rounding, membrane fusion, and syncytia formation were observed at 4–5 dpi, with P0 viral stocks harvested at 50% CPE. As shown in Figure 2G, inoculation of P0 virus into CRFK induced obvious cytopathology: within 24 hpi, cells aggregated and partially detached, with adherent cells exhibiting morphological distortion. By 48 hpi, prominent syncytia formation and widespread CPE were evident across all strains, including cytoplasmic green fluorescence in rFCoV 79-1146-eGFP-infected cells under fluorescence microscopy (Figure 3A). By 96 hpi, extensive cell clumping and detachment dominated the culture. All the recombinant mutant strains can proliferate normally in CRFK, with growth kinetics and growth characteristics comparable to the parental strain rFCoV 79–1,146.

Viral RNA extracted from supernatants was reverse-transcribed, and the genome was amplified in 16 overlapping fragments (Figure 2E). Sequencing confirmed perfect alignment with the cDNA clones, with no mutations or homologous recombination. Indirect immunofluorescence assay (IFA) using a polyclonal anti-FCoV N antibody revealed cytoplasmic N protein fluorescence in the cytoplasm of infected cells (Figure 2F). Western blot detected a ~ 40 kDa band corresponding to the N protein (Figure 2D), confirming expression. In summary, these results demonstrate that the CRISPR-based editing system enables efficient, rapid, and precise modification of infectious cDNA clones. The engineered plasmids retain full infectivity, successfully rescuing recombinant viruses capable of robust replication and exogenous eGFP protein expression. This platform enables systematic dissection of gene-function relationships in FCoV pathogenesis while providing a scalable tool for therapeutic development, and combines high-fidelity genome editing with streamlined workflows, offering a powerful tool for functional virology studies and vaccine development.

### 3.4 Application of the fluorescent reporter virus

The P0 virus strain of rFCoV 79-1146-eGFP was inoculated into CRFK cells and cytoplasmic eGFP expression was observed in infected cells under an inverted fluorescence microscope (Figure 3A), confirming the infectivity of the rescued virus and its ability to express exogenous eGFP, which served as a real-time indicator of viral infection.

To evaluate the utility of rFCoV 79-1146-eGFP for antiviral drug screening, cells were infected at an MOI of 0.1 and co-cultured with GS-441524 serially diluted in twofold steps in complete medium. At low drug concentrations, minimal antiviral activity was observed: infected cells exhibited aggregation, shrinkage, and lysis, consistent with unchecked viral replication. In contrast, high drug concentrations markedly suppressed viral spread, with most cells retaining normal morphology and limited syncytia formation (Figure 3B).

To quantify inhibition, infected adherent cells were trypsinized into single-cell suspensions and analyzed by flow cytometry (10,000 events per sample). While low drug concentrations yielded variable fluorescence signals due to background noise, higher concentrations significantly reduced the percentage of GFP-positive cells and diminished eGFP fluorescence intensity, reflecting dose-dependent suppression of viral entry and replication (Figure 3C). These results demonstrate that rFCoV 79-1146-eGFP serves as a sensitive reporter system for assessing antiviral efficacy *in vitro*, enabling rapid visualization and quantification of drug effects.

## 4 Discussion

Feline coronavirus poses a significant threat to feline health. To date, the relationship and transition mechanism between FECV and FIPV remain inconclusively elucidated. Key gaps in FCoV pathogenesis research—particularly regarding incomplete understanding of genome regulation, viral protein functions, biotype transition, and virulence factors—hinder our understanding of the relationship between FECV and FIPV, as well as the identification of effective therapeutic and vaccine targets. There is an urgent need to establish robust research platforms that advance our understanding of FCoV biology and pathogenesis, while accelerating the development and evaluation of novel antivirals and vaccines.

To elucidate the structural and functional roles of viral genes and their encoded proteins, precise editing of the viral genome is indispensable. In this study, we integrated TAR cloning with the GTR-CRISPR system to achieve efficient genome editing and successfully rescue genetically modified viruses. We demonstrated that CRISPR-Cas9 components in yeast not only target the yeast genome but also cleave exogenous plasmids introduced into the cells. Leveraging exogenous donor DNA and the yeast’s endogenous homologous recombination machinery, DSBs induced in exogenous plasmids were repaired, enabling precise editing of exogenous plasmid DNA. Using the FCoV 79–1,146 infectious cDNA clone plasmid as a template, we introduced site-specific mutations, gene knockouts, and gene replacements to generate five distinct genetically edited infectious cDNA clones. These clones were then used to rescue recombinant viruses. After validation by Western Blot and IFA, we successfully obtained five infectious recombinant mutant strains. Subsequent nucleic acid extraction, RT-PCR, and full-genome sequencing confirmed that all strains matched the expected genetic profiles. Collectively, we established a CRISPR-based genome editing system for FCoV, enabling rapid, flexible, and efficient modification of the FCoV genome.

Coronaviruses are RNA viruses with high mutation rates, yet studying the functional significance of these mutations requires precise genetic manipulation. The genome editing system developed in this study not only advances feline coronavirus research but also provides a powerful and flexible platform that can be adapted to other coronaviruses across species, facilitating the investigation of mutation-driven changes in viral replication, host range, and pathogenicity.

## Data Availability

The raw data supporting the conclusions of this article will be made available by the authors, without undue reservation.
